# Clinical biochemical significance of STAT6, ERG, and miR-647 expression in prostate cancer: Associations with tumor aggressiveness and patient prognosis

**DOI:** 10.5937/jomb0-63731

**Published:** 2026-05-22

**Authors:** Xin Hong, Lin Zhao, Tian Wang, Xiaobing Yang, Changzhen Hao

**Affiliations:** 1 Peking University International Hospital, Department of Urology, Beijing, China

**Keywords:** prostate cancer, STAT6, ERG, miR-647, molecular biomarkers, qPCR, prognosis, rak prostate, STAT6, ERG, miR-647, molekularni biomarkeri, qPCR, prognoza

## Abstract

**Background:**

Reliable molecular indicators that can facilitate early detection and prognosis prediction in prostate cancer are still insufficient. Although Transcription 6 (STAT6), ERG, and microRNA-647 (miR-647) have recently been recognized as key participants in tumor-related signaling pathways, their specific biochemical functions in the context of prostate cancer have yet to be clearly defined. This study evaluated the expression profiles of STAT6 mRNA, ERG mRNA, and miR-647 in prostate cancer tissue and explored their associations with clinicopathological features and patient outcomes.

**Methods:**

Surgical specimens were obtained from 70 patients, including both prostate cancer tissues and their corresponding adjacent non-tumorous counterparts. Quantitative real-time PCR (qPCR) was used to measure STAT6 mRNA, ERG mRNA, and miR-647 expression. The relationships between biomarker expression and clinical parameters - including age, tumor diameter, lymph node involvement, T stage, and pathological differentiation - were examined. Overall survival (OS) and progression-free survival (PFS) were evaluated using Kaplan-Meier curves with log-rank comparisons, while Spearman correlation analysis was employed to determine the associations among the examined molecular markers.

## Abstract (sr)

**Uvod:**

Pouzdani molekularni indikatori koji mogu olaksati rano otkrivanje i predviđanje prognoze raka prostate jos uvek nisu dovoljni. Iako su transkripcija 6 (STAT6), ERG i mikroRNK-647 (miR-647) nedavno prepoznate kao ključni učesnici u signalnim putevima povezanim sa tumorom, njihove specifične biohemijske funkcije u kontekstu raka prostate jos uvek nisu jasno definisane. Ova studija je procenila profile ekspresije STAT6 mRNA, ERG mRNA i miR-647 u tkivu raka prostate i istražila njihovu povezanost sa kliničko-patoloskim karakteristikama i ishodima pacijenata.

**Metode:**

Hirurski uzorci su dobijeni od 70 pacijenata, uključujuci tkiva raka prostate i njihove odgovarajuće susedne netumorske pandane. Kvantitativna PCR u realnom vremenu (qPCR) je korišćena za merenje ekspresije STAT6 mRNA, ERG mRNA i miR-647. Ispitani su odnosi između ekspresije biomarkera i kliničkih parametara - uključujući starost, prečnik tumora, zahvacenost limfnih čvorova, T stadijum i patolosku diferencijaciju. Ukupno preživljavanje (OS) i preživljavanje bez progresije bolesti (PFS) su procenjeni koriscenjem Kaplan-Majerovih krivih sa log-rank poređenjima, dok je Spirmanova korelaciona analiza koriscena za određivanje povezanosti između ispitivanih molekularnih markera.

**Rezultati:**

Nivoi STAT6 mRNA, ERG mRNA i miR-647 bili su znatno visi u uzorcima raka prostate nego u uparenim netumorskim tkivima (P&lt;0,05). Povisena ekspresija ovih molekula odgovarala je prisustvu metastaza u limfnim čvorovima, visem T stadijumu i nepovoljnoj histoloskoj diferencijaciji, dok nisu primećene značajne povezanosti sa starošću pacijenta ili dimenzijama tumora. Pacijenti sa visokom ekspresijom STAT6, ERG ili miR-647 pokazali su značajno smanjeno preživljavanje bez progresije bolesti (OS) i preživljavanje bez progresije bolesti (PFS) u poređenju sa grupama sa niskom ekspresijom (sve P&lt;0,05). Ekspresija miR-647 pozitivno je korelirala sa STAT6 mRNA (r=0,867) i ERG mRNA (r=0,724) (P&lt;0,05).

**Zaključak:**

STAT6, ERG i miR-647 pokazuju izraženu prekomernu ekspresiju kod raka prostate, a njihovi povišeni nivoi su usko povezani sa agresivnijim ponašanjem tumora i nepovoljnim kliničkim ishodima. Njihova koordinisana disregulacija ukazuje na potencijalnu molekularnu osu uključenu u progresiju raka prostate. Ovi molekularni indikatori imaju potencijal da poboljšaju biohemijsku stratifikaciju rizika i podrže preciznije molekularne procene u rutinskim kliničkim uslovima.

## Introduction

Prostate cancer remains one of the most common malignancies in men, and both its incidence and associated mortality have continued to rise in recent years [1]. Advances in molecular biology have enabled the identification of numerous biomarkers closely linked to prostate cancer initiation, progression, and prognosis [2]. Among these, Signal Transducer and Activator of Transcription 6 (STAT6), ERG, and miR-647 have attracted increasing interest for their potential roles in prostate tumorigenesis [3][4].

STAT6 is a key mediator of cytokine-driven signaling pathways and participates in the regulation of cell proliferation, differentiation, and immune responses [5]. ERG is a transcription factor and one of the most common gene fusion-associated alterations in prostate cancer; its aberrant expression has been associated with increased tumor invasiveness and poorer clinical outcomes. miR-647, a microRNA that modulates gene expression post-transcriptionally, has been implicated in tumor-related processes, although its specific role in prostate cancer remains incompletely characterized [6][7].

Accordingly, this study aimed to quantify STAT6 mRNA, ERG mRNA, and miR-647 expression in prostate cancer tissues and matched adjacent noncancerous tissues, examine their relationships with clinicopathological characteristics, and evaluate their prognostic value. By integrating molecular expression profiles with clinical outcome data, we sought to identify candidate biomarkers and to elucidate potential molecular pathways involved in the diagnosis and progression of prostate cancer.

## Materials and methods

### General information

Seventy patients with prostate cancer treated at our hospital between July 2019 and July 2022 were enrolled. Inclusion criteria were: (1) meeting published diagnostic criteria for prostate cancer [8]; (2) pathological confirmation of prostate cancer; and(3) no prior endocrine therapy before surgery. The study was approved by the hospital ethics committee, and written informed consent was obtained from all patients and their families. Exclusion criteria were: (1) comorbidities such as hypertension, coronary heart disease, or diabetes; (2) other malignancies; (3) urinary tract infection; (4) severe dysfunction of major organs (e.g., heart, liver, or kidney); and (5) incomplete clinical data.

### Detection methods

During the surgical procedures, both tumor tissues and their corresponding adjacent non-cancerous tissues were collected and immediately preserved in liquid nitrogen for long-term storage in the laboratory. Concurrently, relevant clinical information - including age, tumor dimensions, lymph node status, clinical stage, and histological differentiation - was systematically documented.

### STAT6 mRNA, ERG mRNA, and miR-647 expression in prostate cancer and adjacent non-cancerous tissues

The differential expression of STAT6 mRNA, ERG mRNA, and miR-647 in prostate cancer tissues versus matched adjacent noncancerous tissues was evaluated by quantitative real-time PCR (qPCR). Total RNA was extracted from freshly resected surgical specimens using a commercial kit (QIAGEN, Düsseldorf, Germany). RNA concentration and purity were measured with an N50 microspectrophotometer (IMPLEN, Munich, Germany), and all samples met quality criteria, with an A260/A280 ratio of 1.8-2.0.

Reverse transcription was performed using a standardized cDNA synthesis kit (Vazyme, Nanjing, China). The resulting cDNA was diluted to equal concentration for all samples to ensure analytical consistency. qPCR was carried out using SYBR Green chemistry (Applied Biosystems, Foster City, CA, USA) on an Mx3005P real-time PCR system (Agilent, Santa Clara, CA, USA). GAPDH was used as the internal reference for STAT6 and ERG mRNA, and U6 served as the reference for miR-647 quantification. Primer sequences were as follows: STAT6: forward 5'-LAGGAGAGCAGGGGAAAGGAAG-3', reverse 5'-LTGGCAGGTGGTGGAACTCTT-3'; ERG: forward 5'-TTATCGTGCCAGTAGCAGGT-3', reverse 5'-GATGTTGACGTCTGGAAGGC-3'; miR-647: forward 5'-CTCAACTGGTGTCGTGGAGTC-GGCAATTCAGTTGAGGAAGGAAG-3', reverse 5'-ACACTCCAGCTGGGGTGGCTGCACTCACT-3'. All primers and reference genes were synthesized by Guangzhou Xingzhi Biotechnology Co., Ltd. Relative expression levels were calculated using the 2-^DD^Ct method, following MIQE (Minimum Information for Publication of Quantitative Real-Time PCR Experiments) guidelines to ensure methodological reproducibility and biochemical reliability.

### STAT6 mRNA, ERG mRNA, and miR-647 expression across clinicopathological subgroups

To further elucidate the biochemical relevance of these markers in tumor progression, STAT6 mRNA, ERG mRNA, and miR-647 expression levels were compared across key clinicopathological subgroups, including age, tumor diameter, lymph node status, clinical T stage, and histological differentiation. These analyses were conducted to determine whether marker expression correlated with established indicators of tumor aggressiveness and to assess their potential value for biochemical risk stratification.

### Survival curve analysis

Follow-up commenced at the time of diagnosis and continued through July 2023, yielding a follow-up duration of 12 to 48 months for all participants. The median follow-up period was 30 months, during which patient outcomes such as survival, death, recurrence, and disease progression were systematically recorded.

### Correlation analysis

Spearman correlation analysis was performed to evaluate the relationships between miR-647 expression and the levels of STAT6 mRNA and ERG mRNA.

### Statistical analysis

Statistic Package for Social Science (SPSS) 22.0 (IBM, Armonk, NY, USA) was used for data analysis. Measurement data were expressed as (±s), meeting normality and homogeneity of variance between groups, and t-test was used. Kaplan-Meier curves were generated to assess patient survival, and comparisons of median overall survival (OS) and progression-free survival (PFS) between groups were conducted using the log-rank test. Spearman correlation analysis was applied to examine the associations between miR-647 expression and STAT6 or ERG mRNA levels. A P value of <0.05 was considered indicative of statistical significance.

## Results

### Comparison of STAT6 mRNA, ERG mRNA levels, and miR-647 expression levels between prostate cancer tissue and adjacent non-cancerous tissue

Expression levels of STAT6 mRNA, ERG mRNA, and miR-647 were markedly increased in prostate cancer tissues compared with the adjacent non-cancerous tissues, and these differences reached statistical significance (P<0.05, Table 1).

**Table 1:** Comparison of STAT6 mRNA, ERG mRNA Levels, and miR-647 Expression Levels between Prostate Cancer Tissue and Adjacent Non-cancerous Tissue.

Group	Cases	STAT6 mRNA Expression	ERG mRNA Expression	miR-647 Expression
Prostate Cancer Tissue	70	0.61±0.12	1.76±0.32	1.88±0.41
Adjacent Non-cancerous <br>Tissue	70	0.23±0.07	0.81±0.11	0.94±0.09
t		22.885	23.489	18.736
p		0.000	0.000	0.000

### Comparison of STAT6 mRNA, ERG mRNA, and miR-647 indicators among prostate cancer patients with different clinical pathological features

Among patients with prostate cancer, STAT6 mRNA, ERG mRNA, and miR-647 levels showed no significant variation by age or tumor size (P>0.05). However, their expression was significantly associated with lymph node metastasis, clinical T stage, and the degree of histological differentiation (P<0.05, Table 2).

**Table 2:** Comparison of STAT6 mRNA, ERG mRNA, and miR-647 Indicators among Prostate Cancer Patients with Different Clinical Pathological Features (±s).

Indicator		Cases	STAT6	t	P	ERG	t	P	miR-647	t	P
Age				0.387	0.700		0.976	0.332		0.435	0.665
	>60 yrs	25	0.62±0.16			1.81±0.36			1.91±0.40		
	≤60 yrs	45	0.61±0.09			1.73±0.31			1.87±0.35		
Tumor Size				1.787	0.078		1.806	0.075		0.563	0.575
	≥3 cm	27	0.64±0.12			1.84±0.34			1.91±0.38		
	<3 cm	43	0.59±0.11			1.71±0.26			1.86±0.35		
Lymph Node <br>Metastasis				4.307	0.000		12.370	0.000		6.566	0.000
	Yes	7	0.79±0.17			2.76±0.49			2.76±0.57		
	No	63	0.59±0.11			1.65±0.18			1.78±0.35		
T Stage				8.680	0.000		6.056	0.000		2.212	0.030
	T1~T2	51	0.54±0.08			1.41±0.21			1.67±0.42		
	T3~T4	19	0.79±0.16			1.89±0.32			1.96±0.51		
Histological<br>Differentiation				5.830	0.000		3.075	0.003		3.219	0.002
	Well-Moderate	40	0.53±0.09			1.63±0.28			1.66±0.41		
	Poorly<br>Differentiated	30	0.72±0.15			1.86±0.33			2.04±0.54		

### Prognostic analysis of STAT6 mRNA expression level in prostate cancer patients

**Figure 1:**
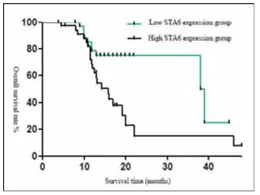
Patient Survival Curve.

**Figure 2:**
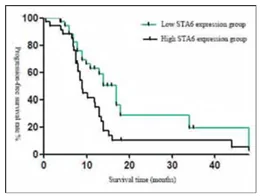
Patient Progression-Free Survival Curve.

Using the median method, prostate cancer patients were divided into high STAT6 expression group and low STAT6 expression group, each with 35 cases. The overall survival rate in the low STAT6 expression group was 25.02%, with a median OS of 39 months, while in the high STAT6 expression group, it was 7.62% and 15.9 months, with statistically significant differences (c^2^=6.240, P=0.013, Figure 1). The progression-free survival rate in the low STAT6 expression group was 19.26%, with a median PFS of 16.9 months, while in the high STAT6 expression group, it was 5.23% and 9 months, with statistically significant differences (c^2^=5.026, P=0.023, Figure 2).

### Prognostic analysis of ERG mRNA expression level in prostate cancer patients

**Figure 3:**
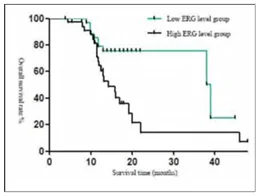
Patient Survival Curve.

**Figure 4:**
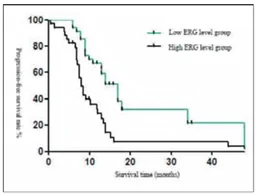
Patient Progression-Free Survival Curve.

Using the median method, prostate cancer patients were divided into high ERG expression group and low ERG expression group, each with 35 cases. The overall survival rate in the low ERG expression group was 25.27%, with a median OS of 39 months, while in the high ERG expression group, it was 7.15% and 14.3 months, with statistically significant differences (c^2^=7.341, P=0.007, Figure 3). The progression-free survival rate in the low ERG expression group was 19.68%, with a median PFS of 16.9 months, while in the high ERG expression group, it was 4.97% and 9 months, with statistically significant differences (c^2^=5.879, P=0.015, Figure 4).

### Prognostic analysis of miR-647 expression level in prostate cancer patients

**Figure 5:**
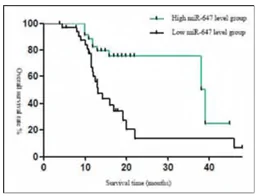
Patient Survival Curve.

**Figure 6:**
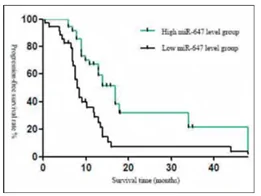
Patient Progression-Free Survival Curve.

Based on the median expression level, patients were categorized into high- and low-miR-647 expression groups, with 35 individuals in each group. The overall survival rate in the low miR-647 expression group was 25.15%, with a median OS of 39 months, while in the high miR-647 expression group, it was 6.92% and the median OS was 13.2 months, with statistically significant differences (c^2^=9.450, P=0.002, Figure 5). In the low miR-647 expression group, the progression-free survival rate reached 21.42% and the median PFS was 16.9 months. In contrast, the high-expression group showed a markedly lower progression-free survival rate of 3.62% and a median PFS of 8 months. These differences were statistically significant (c^2^=11.130, P=0.001; Figure 6).

### Correlation analysis of miR-647 expression and STAT6 mRNA, ERG mRNA levels

The correlation analysis revealed that miR-647 expression is positively correlated with the levels of STAT6 mRNA and ERG mRNA in patients (r=0.867, 0.724, P<0.05).

## Discussion

At present, the clinical management of prostate cancer is largely dependent on conventional clinical assessments and pathological findings.; however, the absence of highly specific and sensitive molecular biomarkers continues to limit early detection and accurate prognostic assessment [8][9]. This has stimulated increasing interest in identifying novel biochemical indicators that reflect the molecular mechanisms underlying tumor progression [10]. In this context, our study examined the expression patterns and clinical relevance of STAT6 mRNA, ERG mRNA, and miR-647, three emerging molecular markers involved in the regulation of oncogenic pathways in prostate cancer.

In this study, all three markers - STAT6, ERG, and miR-647 - were significantly upregulated in prostate cancer tissues compared with matched adjacent non-tumorous tissues, suggesting that they may contribute to tumor initiation and progression. These findings align with recent molecular profiling studies implicating dysregulation of STAT6, ERG, and microRNAs in prostate cancer pathobiology [11]. Aberrant expression of STAT6 and ERG has been reported across multiple malignancies and is often associated with greater invasiveness, more rapid progression, and poorer clinical outcomes. Likewise, miR-647 has been implicated in regulating cell proliferation, invasion, and metastatic potential through modulation of downstream oncogenic and tumor-suppressive pathways [12].

The observed associations between elevated expression of these biomarkers and adverse clinicopathological features - including lymph node involvement, advanced T stage, and poor histological differentiation - underscore their close link to aggressive prostate cancer behavior [13]. Mechanistically, STAT6 primarily acts through activation of the JAK-STAT signaling pathway. In malignant tissues, STAT6 activation has been associated with increased tumor cell proliferation, resistance to apoptosis, and remodeling of the immune microenvironment in ways that promote immune evasion and tumor persistence [14]. ERG overexpression in prostate cancer most commonly results from TMPRSS2-ERG gene fusion, which places ERG under androgen-responsive regulation [15]. Aberrant ERG activity can disrupt chromatin organization, compromise DNA repair programs, and alter cell adhesion and migratory properties, thereby facilitating invasion and metastasis.

miR-647 acts at the post-transcriptional level, binding to the 3' untranslated regions of target mRNAs and thereby reducing protein production through inhibition of translation or promotion of mRNA degradation. In prostate cancer, miR-647 may influence tumor behavior by modulating key signaling pathways governing cell cycle progression, apoptosis, and metastatic potential [16]. In this context, the concurrent overexpression of STAT6 and ERG suggests that miR-647 may act upstream or in parallel to these transcriptional regulators, collectively contributing to the molecular phenotype of aggressive prostate cancer [17].

Our prognostic evaluation showed that individuals with elevated STAT6 mRNA, ERG mRNA, or miR-647 expression had markedly worse overall survival and progression-free survival than those with lower expression levels. These results indicate that elevated expression of these markers correlates with more aggressive tumor biology, increased metastatic potential, and unfavorable clinical outcomes [18]. Furthermore, the positive correlations identified between miR-647 and both STAT6 and ERG suggest potential co-regulation or convergence upon shared oncogenic pathways, such as those involving cell cycle regulation, DNA repair, and cell adhesion [19]. Upregulation of miR-647 may also reflect microenvironmental alterations - including inflammatory cytokines and growth factor signaling - that simultaneously enhance STAT6 and ERG activity [20]. Such coordinated dysregulation may drive synchronous molecular alterations that potentiate prostate cancer progression.

In conclusion, our results indicate that STAT6 mRNA, ERG mRNA, and miR-647 are markedly overexpressed in prostate cancer and show clear associations with more advanced pathological characteristics and unfavorable clinical outcomes. The interactions among these biomarkers suggest a potential molecular axis contributing to tumor progression. These data support their utility as promising biochemical markers for disease stratification and may contribute to future development of targeted diagnostic or therapeutic strategies in prostate cancer.

## Dodatak

### Acknowledgments

This study did not receive any funding in any form.

### Conflict of interest statement

All the authors declare that they have no conflict of interest in this work.

### Contributions

Xin Hong and Lin Zhao contributed equally to this work.
